# Atmospheres of progress in a data-based school

**DOI:** 10.1177/1474474015575473

**Published:** 2016-01

**Authors:** Matt Finn

**Affiliations:** University of Exeter, UK; Durham University, UK

**Keywords:** affect, atmospheres, data, education, futurity, progress, schools

## Abstract

In this article, I seek to extend the geographies of education, youth and young people by offering an account of the significant shifts taking place in contemporary English state education around the production and use of data. I present material from pupils, for whom the changes are putatively made, whose voices are absent in existing educational and sociological literature on data in schools. I do this through an exploration of one specific feature of school datascapes: the use of data to create and maintain a sense of ‘progress’. This is not progress solely as developmental fact, logic, ideology or discourse but as felt. This article draws attention to profound changes to cultures of education that are evinced in relation to contemporary proliferations of data, contributes to theorisations of affective atmospheres in geography and how they come to be known (as a question of both experience and method), and it advances a novel theorisation of progress ‘after the affective turn’.

## Introduction

State-funded schools in England have over the last decade, as part of the rise of an ‘audit culture’^1^ and the embedding of digital technology,^2^ been instructed by successive governments^3^ to give more attention to what data can do for education. Indeed, with policy reflection on, and requiring, change towards schools being ‘data rich’,^4^ it has been said that such changes will bring improvement and foster ‘intelligent accountability’^5^ through evidenced self-evaluation.^6^ This has led to changes in the co-production of data with and about pupils, parents and staff. As yet, this has received little attention from geographers of education, and furthermore, with some notable exceptions, cultural geographers have been slow to engage with the digital.^7^ Nevertheless, concerns about school data have been a long-standing area of interest for school improvement/effectiveness educationalists,^8,9^ and data practices in education and for the state are not, in themselves, new.^10^ Yet, even in this area, there has been relatively little critical empirical research on the experiences and perspectives of actors – particularly pupils^11^ – about this data work of collection/production, management, analysis, interpretation and of maintaining the flows of data which have come to be seen as ‘part of everyday life in modern “learning”/“knowing” organisations’.^12^

In this article, I offer an account of the significant shifts taking place in contemporary English state education around the production and use of data and present material from pupils, for whom the changes are putatively made. I explore an emerging feature of school datascapes: the use of data to create and maintain a sense of ‘progress’ and the affective relations that are associated with these sensibilities. This is not progress solely as developmental fact, logic, ideology or discourse but as felt. That is, a positive feeling in relation to a sense of onward movement – as an increasing mastery of knowledge and skills. I use the term ‘atmospheres of progress’ to describe the occurrence of spatially specific shared senses of progress-making (or the lack of it) that are collective and yet also individualising.

This article is based on PhD research in one school^13^ in an economically deprived area^14^ of the North East of England over the course of a year (October 2012 to November 2013). Retrospectively, for the year in which the study took place, the school was categorised in the top 10 state schools in England (out of 3,500) for ‘adding value’ to pupil exam results – that is, in helping students make the most progress. While this makes the school exceptional, which could raise issues of generalisability, it also allows for exploration of the processes involved through a case study in which these phenomena might be most pronounced.^15^ Its applicability is based on judgements of the extent to which it shows in particularly vivid terms something that is present elsewhere^16^ as suggested by the wider research drawn on here. The research was conducted in several phases to engage with the interplay of the nestled scalar hierarchies^17^ which relate a range of different actors together through data. I turn now to locate this research in the context of geographies of education, and education literature more broadly, and go on to lay out why I think the work of geographers on affective atmospheres is particularly helpful in making sense of one of the modes of feeling that are made, sustained and contested in relation to data.

### Geographies of education, youth and data

Engagement by geographers of education with the rise of data-based education systems has, to date, been limited, and where it has occurred, it has often been conceived in other terms, such as league tables, rankings or grades.^18^ The research presented here most closely connects with that which has explored contemporary education as an emotional project and not only one of the mind.^19^ For while data may be presented as inherently wedded to rationalist, technicist and/or bureaucratic logics,^20^ I would like to suggest that data doesn’t only change or enable particular modes of thought but also modes of individual and collective feeling. These modes of thinking and feeling are not static but, being informed by writings on the ontological status of young people as both beings and becomings, are dynamic. Here, the figure of the Child has more typically been characterised by his or her futurity^21^ as a developmental subject. Whether in relation to the future of the nation^22^ or as a figure which guarantees the meaning of action as for posterity, with the Child as the imagined beneficiary, the absolute necessity of the (‘appropriate’) raising of children is held almost unquestioned.^23^ One of the ways in which that ‘appropriate intervention’ is currently expressed in many countries is through the separation of adult’s and children’s worlds,^24^ with children’s labour being the work of becoming, of becoming adult, of making progress, this being ensured and maximised through compulsory schooling.^25^ Schools take on characteristics attributed to their children: a place of becoming in which the promise of nascent futures are incrementally realised in the present. My contention is schools are places of *making* progress where forms of testing create temporal comparisons (a before and after) that allow for the hierarchisation of difference and change. The profound restructuring that is taking place in advanced capitalist education sectors^26^ is shaped by concerns to maximise and render accountable the productive conditions of schooling.^27^ A proliferation of data is one response. The data in view here typically include records about attendance; behaviour; surveys about attitudes towards learning, teachers and school; pupils’ views on school life, and, as the main focus of this article, records and projections concerning academic achievement. These data are asked to perform two functions which can be understood as being in tension: improvement-evaluation and accountability-monitoring,^28^ with teachers much more supportive of the former function as enabling them to reflect on their practice as teachers and more critical of the latter as surveillance with the intent to punish or shame.^29^ These two functions are reproduced at many nestled scales of interaction.^30^ For example, this proliferation of data enables the ‘fabrication of quality’ and the construction of national and international ‘policy spaces’^31^ and the making of education ‘machine readable’.^32^ While these contributions and the first survey pieces^33^ are instructive, some remain speculative and agenda setting, while those with an empirical basis have tended to focus on the macro-scale with less sense of how the life of data and data-based living are negotiated in detail, in place and in practice. I would like to offer a sense of this negotiated data-based life in school through the following vignette. I go on to take this up in justifying the conceptual work that leads me to the term ‘atmospheres of progress’ which I unfold through the rest of the article.

## Finding yourself in the spreadsheet and feeling good

**Figure 1.**
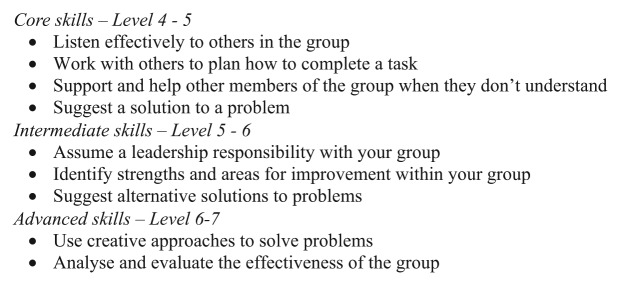
Assessing Pupil Progress (APP) board – communication and problem solving skills.

The pupils gather to the teacher. Now in secondary school^34^ they have been doing team building exercises in Physical Education classes. The teacher hands out two copies of a printed spreadsheet and the pupils take them to the floor nearby. Sprawled out, lying down on their front, others kneeling, the pupils trace out together their ‘levels’ gathered around the pages. Criteria within each level are marked as achieved or yet to be met, and every pupil’s attainment data in the class are included. There is an informality about the postures adopted and the relaxed, even animated communication. ‘I’ve got a [level] 4 in this one’, says one young person. ‘Yeah but you’ve got the 5 for this part already’ another replies.^35^ The tone of the communication is light; the only overt antagonism occurs when one pupil wants to turn the page over to look at their entry. ‘Read them [your targets] so you know what you need to show on Friday’, the teacher calls. Pupils move to an ‘Assessing Pupil Progress (APP)’ board on the wall and again help each other look up on the board what they each need to do (and show they can do, see Figure 1). APP boards for different subjects are present throughout the school.

The numbers on the spreadsheet locate the pupil’s current evidenced level of achievement and tell them (with reference to the boards) what they need to do next. This is learning as progress through defined levels of knowledge learnt and skills demonstrated. I see spreadsheets of various kinds elsewhere too, put up in other lessons on the electronic whiteboards. Groups of young people go up together and trace out each other’s levels. They help each other make sense of what is done as they look at the filled entries for assessments and what is still possible as they see the empty spreadsheet columns and rows. They encourage and console each other. These, as some of the most visible moments of encountering data, are taken up to provide occasions for sociality, even the strengthening of friendships through relations of care. The young people, a surprising amount of the time, appear to come out of these encounters with data feeling good – irrespective of their level, most of them are making progress. The sense of feeling is collective – as almost passed around through touch, looks and laughter between pupils and also with the teacher – at the same time as it is individualising with respect to locating pupil performance as a feature of her self, shorn from relations. This sense is visible in a pupil’s demeanour and bodily comportment and the tenor of their interactions with others, but it is also beyond any one pupil as some kind of collective good feeling ‘in the air’. It is, as I go on to discuss, something atmospheric.

### Encountering atmospheres

To make sense of this vignette and other moments like this, in which data are taken up in learning spaces in the school, with reference to concepts like emotion or school ethos seems to me to be inadequate. While emotion is all too easily personal and individualised, ethos is also too easily imagined as collective but free-floating from socio-material and historical circumstances.^36^ Emotions are clearly experienced by the young people in relation to data, and yet, it would be a mistake, I believe, to reduce encounters with data to the biographical, as private feelings evinced in relation to a digitally reflected mirror of the self-in-bits-and-bytes. While Bragg and Manchester^37^ argue for an understanding of school ethos that is more consonant with the way I am making sense of this mode of feeling (as interpersonal, material and social and continually negotiated), a key difference is that ethos retains a sense of relative obduracy as something which ongoingly characterises ‘the school’ as a whole. In contrast, I want to name something which is more fragile, more fleeting and operates in ‘pockets’ or spheres which emerge and envelop members of the school in some classes and not others. And, in the context of atmospheres of progress, this mode of collective and individualising feeling is something which can be made and sustained and falter in the same lesson with the same pupils and teacher in relation to the production and use of data.

For this reason, I turn to the concept of affective atmospheres which geographers, among others, have found interesting, not least in part, because of the way it holds a ‘series of opposites – presence and absence, materiality and ideality, definite and indefinite, singularity and generality – in a relation of tension’.^38^ There is an ambiguity to atmospheres, in both the meteorological sense and of those affectively sensed,^39^ that seems to make them interesting empirically and theoretically. Indeed, it is the multiplicity of the referent for the term atmosphere^40^ that allows so many to become *attuned*, to use Stewart’s language,^41^ to so much that has intensity and force in the world. Yet, whether one says ‘attune to’ or ‘attend to’ or ‘apprehend’, it is still assumed that there is some*thing* to be openly disposed towards. While Bissell cautions those attuning themselves to affective atmospheres^42^ that such atmospheres should not be ‘reified as a “thing”’,^43^ it is precisely this ‘thingness’ to which I would like to pay attention.^44^ As a thing that can be worked on and worked at, known intensely and with particularity, as something to which people attribute casual power. While it is perhaps more straightforward methodologically to attend to atmospheres (as-a-thing) themselves, it is also appropriate to attend to the range of bodies (human, discursive, non-human) from which atmospheres may be said to emanate.^45^

Although attention has been drawn to the spatiality of atmos-*spheres*,^46^ with the possibility of a centre and circumference, however, indefinite or unstable, there appears to be a reticence more broadly to consider atmospheres as bounded. This would be to account for the experience of atmospheres, not so much as backdrop,^47^ or the ‘hum of the ordinary’^48^ but as suddenly and powerfully encountered as with the crossing of a boundary. To pass from one sphere to another or to feel oneself held inside or outside of a collective affect. This is to name experiences as discontinuous even if they are theorised as continuous but with changing intensities, not so much created as recomposed differently. The apparent power to change or ‘kill’ the atmosphere can come with the same startling rapidity, where someone’s mere bodily presence ruptures the collective interpersonal sensibilities as with the ‘killjoy’^49^ or the ‘party-pooper’. Although I would suggest that geographers need to make room in their accounts of atmospheres for this kind of experience, it is not the case that these experiences are set apart from the material elements of the world (as if immaterial), or the lived experiences and socio-economic histories of those persons involved (as if ahistorical). They are not spontaneous as they may feel.

So as Ahmed writes,

“Let’s take this figure of the feminist killjoy seriously. Does the feminist kill other people’s joy by pointing out moments of sexism? Or does she expose the bad feelings that get hidden, displaced, or negated under public signs of joy? Does bad feeling enter the room when somebody expresses anger about things, or could anger be the moment when the bad feelings that circulate through objects get brought to the surface in a certain way?”^50^

Ahmed’s language of surface implies a delineation between what is apparent and that which is present but hidden. We could understand this to refer to the potential of bodies to affect and be affected which even when actualised may not be evinced. In other words, these various bodies may be affected but have the capacity to hold hidden the circulating feelings until a moment of eruption or encounter. This suggests that feelings through objects (such as the material presence of data in the school) may have a history that is not immediately visible. Such atmospheres would not then be spontaneous, discontinuous experiences for a perceiving body even if that body were to account for that experience in those ways. This presents a methodological problem, however, as to how the expressivity of an atmosphere comes to be felt and known and named in spite of such indeterminacy. Indeed, if one is to think of atmospheres in relation to the socio-material histories of both people and their data with histories which are not immediately visible, familiar methods might need to be taken in less familiar directions.

### Following the data – feeling, knowing and naming atmospheres

For this project, I brought together four means of following the data that were produced and circulated within the school and sought through these methods to attend to the atmospheres which I had felt enveloped by in particular spaces and at particular times. Since there is very little empirical material which describes the ways that data-work and talk about data feature in the classroom and life of the school, I observed 11 days of lessons (55 hours) in October and November 2012 and took notes based on my observations and interactions with staff and pupils. I saw lessons in every year group.^51^ I sought to follow the material appearances of data in the classroom in speech and on walls, electronic whiteboards, computers and classwork books and written tests. I sought to attend to the atmosphere(s) of the lesson through paying attention to a range of interactions. I watched pupils’ bodily comportments noting how people stood, sat, slumped, moved around and used their bodies in individual and group learning activities. I noted the signals of smiles or downcast eyes. I heard the sounds of the classroom from the huffings, raised voices and throwing down of school bags in frustration and anger to the ‘bright’ tenor of many voices animatedly talking and reflecting together or the ‘deep’ silence of stilled bodies in thought. Collectively, these contribute to a sense of collective feeling that I too was enrolled in and tried to rationalise in and through my body. Drawing on previous experience of work and research in schools, I made sense of these as atmospheres of progress in that they combined those interactions and comportments which are commonly associated in this raced, gendered, classed context as evidence of engagement and positive feeling with a sense of this resulting from feeling improvement as a movement through levels.

However, my own experience and interpretation of these atmospheres may differ wildly from those of the pupils and teachers, due to my own positionality as neither teacher nor pupil but also in terms of my own biography. I therefore interviewed pupils and staff. I interviewed 19 Year 10 and Year 11 (14- to 16-year-olds) pupils in 13 interviews that ranged from 30 minutes to an hour. Having outlined the project in an assembly, pupils contacted me via the school email system. They could be interviewed individually or with a friend, and in three of the interviews, there were two or more young people present. CDs of the interviews were offered to the pupils. We talked about how they thought and felt about some of the specific presences of data in the school, and I asked them about things I had observed. They spoke in ways which articulated some of their experiences of data and in the individual and collective feelings associated with them that were not apparent in lessons. This and some activity based prompts about their own data and things they and ‘the school’ valued elicited rich data with a strong dimension about how they (make) sense (of) the atmospheres of the school and sometimes exploit and sometimes struggle to negotiate their interactions in different classes and with different teachers.

Similarly interviewing teachers, we talked about how the school had changed over the time they had been here and about the use and limits of data in the classroom, paying attention to the roles they thought that data play in schools and their role in managing this. This was particularly pertinent to atmospheres in relation to their skills in classroom ‘behaviour management’^52^ which can be understood as a form of socio-spatial affective orchestration. I interviewed 12 teachers at various career stages and subject areas for an hour to an hour and a half. I also attended Continuing Professional Development (CPD) training sessions for staff. This contributes to and extends the research by Kelley et al.^53^ already done based on a national survey of staff and some interviews.

The final part of the project involved working with pupils from the ‘Student Voice’^54^ group over 8 weeks to develop participatory research projects that they chose and conducted. While not directly related to data, this part of the project was part of my ethical commitment to pupils at the school. They chose to research two aspects of pupil–teacher relations: how teachers relate to pupils in different year groups differently and how pupils experience different lessons through the school day. They presented their research to staff and then to students and staff at DurhamUniversity’s Geography Department. These too had a strong atmospheric component and in part explored what it might mean for pupils to exercise more apparent control in determining the conditions of their affective experiences of school.

These methods together sought to attend to the life of data and of people’s experience of data-based living, in particular, thinking about the modes of feeling – the affective atmospheres – made, sustained and contested in relation to data. In this article, I draw together elements from all of these phases of the research alongside policy documents. Before I turn to staff and pupil’s experiences of data as implicated in these processes, I would like to make an argument for why ‘progress’ has taken on a new significance in the English state education system and why understanding this is necessary in accounting for some of the proliferation of certain kinds of data in schools.

## The turn to progress

**Figure 2.**
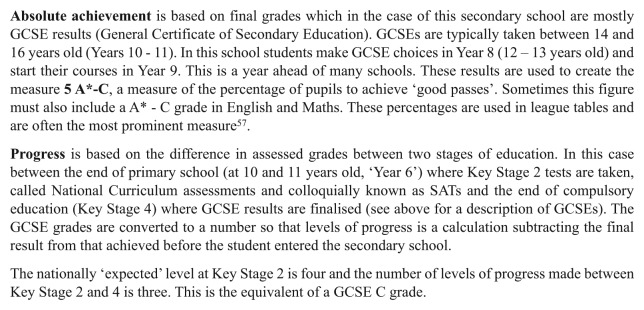
Achievement and progress.

Progress in education has taken on new significance in the English state education system in recent times, starting under the last Labour government and continuing with the current Coalition government.^55^ A key question of educational accountability and judgement making is how to separate the work of the teacher and school-as-a-whole from that of the pupil when teaching and learning are co-produced. A shift was seen under New Labour^56^ from judging the school on the basis of absolute achievement to the progress made while at the school (see Figure 2).

Llewellyn writes,

in New Labour’s first white paper they state that ‘school performance tables will be more useful, showing the rate of progress pupils have made as well as their absolute levels of achievement (DfEE, 1997, p. 6). Specifically they will ‘focus more on the progress made between different stages’. (DfEE, 1997, p. 26)^58^

The introduction of progress data (and it is important to remember this is an operationalisation for a particular idea of progress) is justified as a question of utility. The problem assumed is that the number of General Certificates of Secondary Education (GCSEs) a pupil attains and at what grade did not give any indication of where they had started out when they entered the school. Perhaps they came in at low levels of achievement and made rapid progress, perhaps they came in with high prior levels of achievement and school had very little effect in helping them improve. Furthermore, sufficiently significant proportions of the variation in GCSEs grades are explained by factors that are outside of the school’s control, making them unhelpful in assessing the role any particular school has played in a young person’s education.^59^ The addition of progress data promised to remove the differentials in prior attainment and to isolate the amount of progress made while at that school. This is meant to stop the rewarding in league tables of some schools based on the cultural capital of their middle class pupils and stops other schools being failed on the basis of the structural disadvantages which affect the pupils they teach. Conversely, the shift is held as allowing the idea of equality of opportunity (and outcome in a very specific sense) to be held as all pupils are expected to make the same levels of progress irrespective of their socio-economic position or family circumstances. Furthermore, it allows for the putative freedoms of schools from prescriptive methods dictated by central government while increasing centralised control based on the specification of which outcomes are to be held to be valuable.

In line with this, the Teachers’ Standards document issued by the Coalition government’s^60^ Department for Education continues this theme, and selected parts outline that a teacher must

Promote good progress and outcomes by pupils

be accountable for pupils’ attainment, progress and outcomes

guide pupils to reflect on the progress they have made and their emerging needs

Make accurate and productive use of assessment

make use of formative and summative assessment to secure pupils’ progress

use relevant data to monitor progress, set targets and plan subsequent lessons

From this, we understand that teachers are no longer held to be responsible merely for teaching, the ‘input’ – to use that language – but to promote, secure and be accountable for progress, that is, for ‘outcomes’. But how may a pupil be said to have made progress? How is this known, indeed, produced as knowable? What are the conditions of possibility for ‘progress’?

Producing progress requires many things (and the following is not exhaustive): the cultivation of professional judgement and methods for standardising this judgement nationally: teacher knowledge and skills in data handling and analysis, reconfiguring and fixing knowledge and skills into hierarchical (stagist) national curriculum levels which pupils can be shown to have achieved. Furthermore, database software and/or spreadsheets are used which calculate the levels of progress made (see Figure 2).

One of the sites for shaping the collective knowledge and skills of staff that I observed was an after-school CPD session on the use of data in the researched school. One of the teachers leading the session said which page of the spreadsheet staff should pay most attention to:

For me the best sheet to be looking at is progress; ultimately as a teacher that’s what you’re judged on.

Although a pupil making three levels of progress between 11 and 16 years old can be said to be making nationally expected progress, four levels of progress is what all the staff are expected to promote within the school in which I researched.

An example of the reconfiguration of knowledge is that of vocabulary in English lessons. Schools which seek to operationalise this idea of measuring progress in usage of English language might make a list of words categorised into levels: some are level 4, some level 5, others level 6 and so on. A comment to a pupil who is said to be working at level 4 might be to try and use more level 5 words. The pupils know what level they are on and what they are working towards, and because of the widespread use of self-assessment and peer marking, they may internalise systems of judgement through marking their own and others’ work.^61^

In this way, a concern for progress is bound up with modes of measuring, that is, producing, progress. This follows alongside the discursive shift from *teaching* being the proper focus of teacher’s efforts to the issue of whether *learning* is actually taking place.^62^ In ‘learner-centred’ education,^63^ teachers are made responsible for producing learning – that is, they are made responsible for producing a very specific form of enumerated progress,^64^ separated^65^ from class, ethnicity, gender and family structure and circumstance. Even if everyone cannot achieve the same outcome, all should make progress and at least three if not four levels or more. Ofsted, the Office for Standards in Education, Children’s Services and Skills, which inspects the majority of England’s schools, expects that outstanding teaching is that which ‘over time teaching is enabling almost all pupils to make rapid and sustained progress’.^66^ Lesson observations in schools as part of ‘quality assurances processes’ shift from looking at what the teacher does to finding out what the children have learnt. A proliferation of data about pupil learning is the result. The data-based school of the title is not just one in which data are made about pupils but also one in which decisions are made of the basis of these data. The teacher is not, as conventionally held, a transmitter of information (or in Frierian^67^ terms ‘a banker’ of a static body of knowledge) but a data producer and analyst who enrols the child as the same – as a social scientist of their own learning ability, achievements and life trajectory. The school might not be thought of as an ‘exam factory’ where high-stakes public testing is in view but a ‘data factory’ as everyday practice. Data become one of the elements that allow progress to be known (or rather produced) as such. Evidencing a ‘then’ and a ‘now’ and ‘not yet’ enrols pupils’ past, present and future selves and bundles them together: cognitively, as a story of improvement for public accountability and affectively as a technique for pupil and staff motivation.

This language of business management is picked up by a head of department at the school:

I’m actually saying [to my departmental team] these are our deliverables, you know, it feels like I’m at Tesco’s. And I’m saying you know we must sell all 30 of 30 pallets of strawberries today because they’ll go out of date. That’s where I feel I am now and that’s just twelve months Matt. Each year I think I’ve honed me skills a little bit more. I’ve became more comfortable with what three levels of progress meant, I became more comfortable what nationally we’re measured against and that comes with time and experience.

Teachers become those tasked with delivering progress for and with young people. This project is something that, as I suggested earlier, requires skill, but also knowledge in the honing of professional judgement. While one mode of this is dehumanising – the pupils are ‘pallets of strawberries’ – as I will argue elsewhere, there are countervailing discourses, particularly around care,^68^ which operate with very different logics.

Delivering progress in schools, or rather producing it, has come to be dependent on the emerging everyday practices of making and using of particular forms of data. This has run alongside the reshaping of the roles, competences, knowledge-curricula and governance of education spaces.

Progress, it can be noted, has more typically been understood as a story about time associated with modernity. The story assumes that there is a universal linear trajectory to history where onwards is upwards.^69^ Progress is read as varying spatially and as originating in certain places (and with certain people) and moving beyond these bounds being shared through the spreading goodness of civilising missions. Where the story resonated with stagist evolutional theories of human development (and child development^70^), it figured strongly in colonial (and neo-colonial) imaginaries and has been strongly critiqued.^71^ Progress as good change is held, in this view, to predominate through the spread of ideas which bear the burden of European thought. For Chakrabarty, these include ‘citizenship, the state, civil society, public sphere, human rights, equality before the law, the individual, distinctions between public and private, the idea of the subject, democracy, popular sovereignty, social justice, scientific rationality’.^72^ Progress has therefore been thought of as ideology, discourse and logic – as a mode of thought. I argue that this occludes the possibility of an affective dimension of progress where pupils visibly demonstrate and also describe making progress as feeling good. Yet, the conditions under which these atmospheres of progress can be made and sustained are volatile. Stabilising the turbulence and fragility of these atmospheres which enrol data in the production of collective and individualising feelings of progress therefore becomes a critical act by staff and sometimes pupils themselves.

## Maintaining an atmosphere of progress

While the level of specification offered in curricula and the confidence with which teachers ‘level’ work by pupils could lead one to see this progress data as settled, solid and static, that would be a mistake. There is fragility to the conditions by which the school may be produced as a place of collective progress – both for pupils and for ‘the government’ or Ofsted – and teachers are highly sensitive to this volatility with their reputation as a school and ability to position pupils for the future at stake. One of the Deputy Heads of the school said,

So they’re only just telling us now how exactly they’re going to measure our performance in 2014. Now the kids who leave in 2014 started their options in 2011. So they’re well down, there’s very little we can do. So you’re trying to second guess what the government is going to do, you’re trying to meet the requirements that they are going to impose on you as well. . . . You’ve got to be continuous reading what the politicians are saying and what they’re obviously pointing at and try and adapt but you’ve got to put the kids first.

Staff find themselves increasingly in a position where they feel obligated to serve the data (and the school’s reputation) in ways that could worsen pupil outcomes in the longer term. What maintains the school as a place seen to be making ‘the right kind’ of progress for state accountability structures is something that may not maintain for pupils a collective sense of progress-making. This requires a certain kind of attunement to the moods of politicians concerning the direction of change. Sometimes, the priorities align, but at other times they diverge, as a head of department at the school reflects:

At the end of the day it’s that balancing act of actually the data’s the data and Ofsted are Ofsted but there’s a child in this and what’s best of the child isn’t sometimes best for the data. So [child’s name] is a classic example where I’ve made a call where he comes first, not my data. And it’s tough and you can see I’m taking a hit there of 1 but I think it’s manageable and I think he comes first.

The situation is presented as something to manage that requires decision-making and involves conflicts of interest. Here, what serves the data (and by extension the priorities of Ofsted) is not that which best serves the interests of the child. To protect the child from the further expectations of government in terms of the progress the child will make is what (counter-intuitively) the teacher believes is necessary to maintain a sense of existing progress that keeps the child engaged in education. This is contentious, and various adult actors disagree about what it means for the ‘child to come first’ or for the child’s best interests to be served. Of course, the possibility is raised of what happens when the ‘hit’ becomes unmanageable.

Given these potential conflicts of interest, I was surprised then that in all the time I spent in lessons and in the interviews, the data were not often overtly contested. Below I offer one example in which the attempts to maintain and hold stable an atmosphere of progress in relation to school data were less effective:

A teacher in a foundation (‘lower ability’) English class reads out to the class pupil’s targets, ‘it’s an aspiration’, she says, ‘it’s what I think you possible should be aiming for’. Down the list she goes reading out the targets and the grade they are ‘on course for’. I hear pupils says, ‘congratulations’, genuinely meant, ‘I don’t want to know’ from another. ‘Same as me’. ‘These could change depending on your work’ the teacher reminds, ‘yeah they’ll go down’ one pupil chimes in. The same pupil keeps talking about failing, ‘I’m going to fail’, goading the teacher into disagreement and encouragement about what he could achieve.

Later the class has completed a two part task with the pupils and they are now peer-marking. She tells them to compare their marks this week with when they did the task last week, many had gone from 3 out of 7 to 6 or 7 out of 7. She drew attention to this and gave praise as to the progress they had made. But one pupil calls out that they couldn’t have got more than three last time because they were only told and given instruction on how to do the first part whereas this time they had done both parts.

The teacher has understood that she must generate a sense that progress is being made, with the pupils and/or for the approval of a school inspector. However, she does so less artfully than other staff and in a way that the pupil perceives to be based on an unfair comparison. The lesson felt ‘flat’ after that moment, pupils were listless, bodies low in the chairs and with little eye contact with the teacher or each other. By contrast, most teachers consistently (and more effectively^73^) worked to manage these individual and collective affects around these encounters with data. In a year 7 geography lesson, assessments were returned and a sense of dismay passed between pupils. Eyes widened and some shock was registering. The collective feeling had moved from anticipation of the results to a ‘loss of heart’ and the possibility of protest from some who had been used to higher marks in primary school. The geography teacher quickly interjects when he perceives a shared sense of dejection:

Don’t get disheartened as there are 4 years [to go] but you’re in the first term of year 7. You’re not expected to be there yet. Mozart & Einstein probably wouldn’t get their target grades yet.

Irrespective of what Mozart and Einstein might have achieved if only they had been able to benefit from these year 7’s target grades, teachers sense the need to maintain circulations of confidence to keep young people enrolled in the process of data creation which would allow for progress to be made and felt as having been made. The threat to an atmosphere of progress in which students feel themselves to be ‘on track’ was dealt with promptly by the teacher. This occurrence was neither a set of individuals having entirely separate emotional responses at the same time nor an example of a school’s pervasive ethos at work, but a particular response to a collective sense that individualised pupils and yet also was collectively experienced, interpreted and responded to by the teacher. The sense of it ‘in the air’, as potentially eruptive and certainly as enveloping me, as someone who hadn’t taken the test, was palpable. The shared sense of progress-making and individual and collective good feeling that accompanies this is reliant on data and the associated technical and emotional judgement and management of staff. And, as I have suggested, not all members of staff are equally effective in maintaining an atmosphere of progress. The agency of data is not pre-determined but highly contingent and its effects dependent on the means by which data are interpreted. I continue to explore this theme in the final section where I specifically consider pupils’ experiences of progress data through their language of ‘push’. I move from considering atmospheres of progress specifically to some of the affective relations that emerge in association with these atmospheres.

## Progress and push

**Figure 3.**
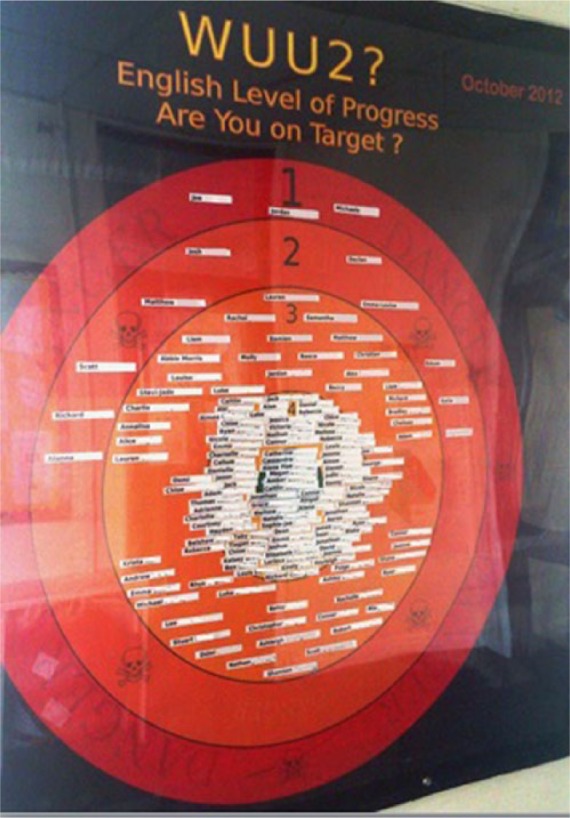
WUU2 boards – What (are) you up to?

Dave is in his final year of this school. He likes to help people and he says that is why he volunteered to be interviewed. He is proud of his home town. Despite this, Dave has been in trouble and nearly removed from the school to alternative education. He is feeling more positive about school, but it has been difficult for him to work out, in his words ‘who’s who and what’s what’. He finds himself having made only two levels of progress in English since the end of primary school, compared with the three levels that are the nationally set expectation. It is important to note that the expected distribution is not the Gaussian normal curve with which educationalists are familiar. The normalisation function is operating differently. Here, all students are expected to make the same minimum number of levels of progress (three) or more, irrespective of their starting point. These data are put on display and have a material presence in lessons on electronic whiteboards, on exercise books, report cards and here in the corridors (Figure 3), in this case by the canteen where students queue for lunch. The figure shows a set of concentric circles each representing levels of progress (from 1 to 6). Each year 11 pupil’s name is placed in the circle of their respective number of levels of progress as in October 2012.

**Figure 4.**
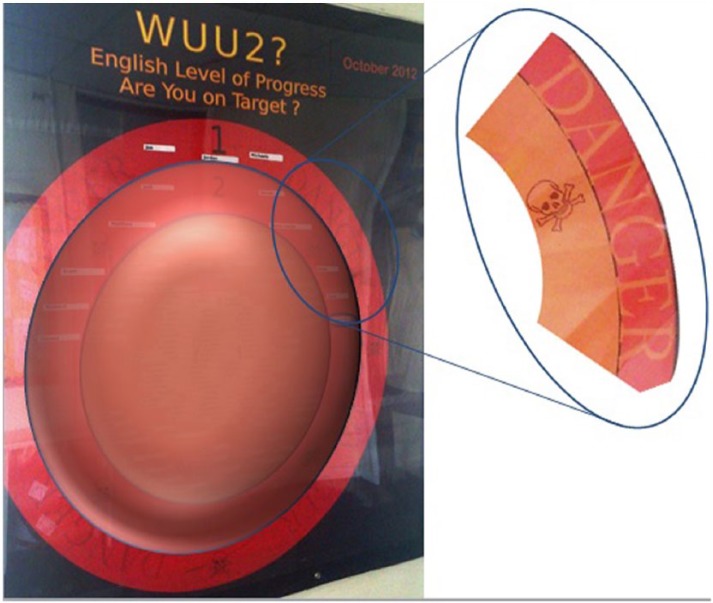
Outside the sphere.

This practice of displaying data is rendered normal in part because of the peer marking I mentioned above; many pupils know each other’s levels already. To add another dimension to this display, I would like to suggest he not only finds himself over a line but outside of the affective sphere of adequate progress (Figure 4). He has made progress but not enough. He is in another sphere in which the outer ring contains the word ‘Danger’ repeated several times alongside images in the outer two rings of a skull and crossbones. What is signified? This sphere is one of danger, of threat and being subject to interventions to try and get him back on track. And what is meant to be at risk here? Progress, life chances, aspiration?

Dave is positioned differently on different boards, but when I ask him about them, he displays resignation about his positioning by them:

I’m not bothered what people think about me, to be fair and whatever I’ve got, that what’s I’ve got. Fair enough. At that target thing, it’s just to show who’s the brainiest and who’s not I reckon. I’m not a big fan of it because I’m always at that end instead that end (he indicates with his hand in the air an outer circle, the edge, rather than near the middle).

I ask him why he thinks that the teachers use these boards:

Just to show people where they’re at so they know if they need to stick in more or they can relax a bit and that’s why I think, I’m not sure. I could never understand the school’s logic.

For Dave, along with many other working class lads,^74^ the school is a confusing place, operating with a logic that is other to himself. The data speak to him of the disposition he may adopt as to whether more or less effort is going to be asked of him. Maintaining engagement and making sure that the data will result in motivation requires significant labour, and being outside this affective sphere renders one subject to intervention. This is a place where staff work to try and pass on and re-mobilise feelings of confidence, and through aspirations discourses, hope, the lack of which, is meant to be part of the problem.^75^ This kind of intervention is quite consciously done and its performative effects well known to staff. The head teacher reflects,

I like data, so I’m all for it and it’s served us well here as a tool to motivate pupils, and staff and the school but when the data is going well it is an uplifting thing. That’s why most people are happy for it to be public in this school because the data is very positive data and that has a cumulative effect over the years. When the data starts to slide, as it will under Mr Gove’s new ideas,^76^ for a school like this it will. His idea of curriculum, of what children study, is not appropriate for many of our pupils at all and they will underachieve because they can’t achieve what he’s expecting them too. That would be a different thing and the data will become an issue again.

Data are seen as having the affective quality of buoyancy (for many though clearly not all), but this quality is contingent on the policy decisions which effect schools differently because of the relationship between a school, local people and post-mining landscape and the nature of the curriculum and the classed values and knowledges assumed to be of import.^77^ Figure 3 is a source of pride for the head teacher because it shows that the majority of pupils were making greater than expected progress and many have made exceptional levels of progress. The use of data here is strategic but ambivalent. I asked her whether using data publicly in this way were to be more demotivating for more pupils whether it was something they would reconsider. The head acknowledges, ‘Yes the WUU2 boards would go’. The data which are in part constitutive of atmospheres of progress could in the future undermine the atmosphere the head is seeking to maintain. It is a contingent practice.

All these data do not only have material presence through the school but are also made present in language. One of the ways that the changing role data plays in maintaining these atmospheres is reflected and reproduced through ‘data-talk’ both from teachers and pupils. The head teacher again reflects,

So about three or four years ago we noticed a difference in the kids. When you listened to their conversations, and as a teacher you can’t help but do that, the conversations had changed. They were much more about what they were targeted and I’m going to get, I’m working towards a C, I think I might be able to get a B in that. I need 5 grade As to get into wherever. I know my deputy who’d been here a long, long time, to her it was a tangible difference in the children thinking about what they were forecast, what they were targeted for, what they needed to get. That had never happened in all the time here. I don’t think that’s unique to here . . . the data drive from Ofsted and government has made it happen.

This comes through in the language used by pupils in interviews. Staff tended to talk about progress, whereas almost all of pupils talked unprompted about ‘push’. So when taking about the technique of ‘aspirational targets’, Dave feels something different:

It’s nice to see they’ve given, like, that’s what you should be aiming for, so like try and get this. It does give you more, uh, . . . what’s the word, . . . motivation, to get that but to get that level. Instead of just sitting back, oh, cos if they give you, like, a low level you just think, what’s the point, no point in doing this?

While Dave might be outside the sphere of adequate progress and so doesn’t share in the good feeling of his peers, when it comes to targets, as judgements of what Dave could achieve, a different set of feelings is called forth. I ask, are there any times when you think it’s not been a motivation?

Personally, not really because I always like, I always try to push, see what I can actually do.

And although he thinks that there are more upsides than downsides to targets like these,

‘there is some downsides when you just like cannat be bothered and you’re like, just they’re pushin you, pushin you and you’re just like, “I cannat”. You cannat keep up and that and you’re just tired, but you get over it’.

An atmosphere of progress then, as described by pupils, is one of push, of movement through pushing yourself and through others pushing you. It also draws in senses of motivation, achievement, pride, despair, boredom and tiredness. Dave articulates the making a decision of the will in sitting back and the feeling of emotion in not being bothered and an inability to ‘keep up’ with the pace of learning. Although others expressed a similar confusion and surprise to Dave about the logic of the school in the setting of targets and whether they are achievable, I should stress that when most of the pupils interviewed spoke about ‘push’, they did so in a positive way.

For Jeff, also 16, accepting this use of data and the concomitant ‘push’ that comes with it is justified by the outcome and the future freedom it offers. While now you have to do particular things ‘then you can just do what you want’:

I’d say that [the school] does help yuh, cos it does push yuh to get the best grade you can so then when you come to the decision to go to college or to sixth form or apprenticeship or whatever, then you can make that decision freely. So you got the grade you needed and then you can just do what you want.

For Elena, the data allowed her access to a slightly different kind of movement and push:

Yeah because, like, they’ll notice if you’re doing well . . . The new teacher realised how well I was doing and how easy I found the work and I actually got pushed up to a higher class, so I wasn’t just sitting there doing easy work. I can actually now do harder work to challenge myself.

Her data had changed sufficiently that she was now not being able to make progress with the level of work available in her class and so gets ‘pushed’ up to a different set. She experiences educational movement in contrast to the stationary who are left ‘just sitting there’ outside the sphere of progress as effortful movement. She feels positive that her achievement is recognised and her journey of progress can continue. In the same interview, her friend Nicki feels less positive. She achieved high Scholastic Assessment Test (SAT) levels for science at primary school but because of the pathway^78^ she was placed on, she is being asked to ‘bank’ a Business and Technology Education Council (BTEC) science qualification. She will then go on to spend a year on GCSE science which ‘because we haven’t had no practice at exams it’s going to be twice as hard for us’. Nicki’s critique is of a BTEC which isn’t ‘worth as much as GCSE’ and is ‘not challenging at all. It’s easy’. She feels, on the basis of her previous grades, it is unjust that she is insufficiently pushed relative to their peers – that her ‘educational movement’ is not requiring her effort. Nicki is held outside of the spheres which would allow her to feel that she too is able to make the kind of progress she would like based on the credentialing data that is most valued in the English education system.

To be an object of the attention of teachers may be unwelcome, but for some, to not come to the attention of teachers and not be subject to intervention is worse – it is to believe that the school isn’t interested in your progress. And, this is not without warrant for the tactical approaches some teachers take do imply uneven geographies of push (and attention^79^).

In one of the CPD session about data use, one member of staff describes looking at those at the boundaries of grades or levels. She asks, is the child on a D+, in which case it’s ‘worth investing the time’, or a D−? Furthermore, ‘if there are lots of D+ who are you going to give them time to?’ Importantly, this is not just focused at the C/D borderline. Another example was given of a pupil who was making three, four or even five levels of progress in all of her other classes but only two levels of progress in one subject, and the teacher says, ‘I know I’ve got to invest time in her’. In this new regime, it is *not* those who are furthest from a ‘passing C’ grade who are given less attention but those who are furthest from *any* next level of progress. Previously, the limited attention of a teacher might have been strategically focused on those at the C/D borderline to ‘get pupils up to’ what is considered a pass who count towards the school’s A*-C measure for league tables.^80^ Those above weren’t pushed and below were written off in this story and received less attention. With the introduction of progress data, the school becomes accountable for pupils making progress across the ‘ability range’. However, the ‘push’ is still given unevenly. The atmospheres of progress are maintained through data and visualisations of that data to try and promote general effects for all pupils. However, there is unevenness to the intensity of the techniques used to (re)mobilise these atmospheres around particular pupils and at different times.

## Conclusions

In this article, I have drawn attention to profound changes to cultures of education that are evinced in relation to contemporary proliferations of data. I have argued that state schools in England are seeing a shift from a focus on absolute achievement to progress and from a focus on improving teaching to evidencing effective learning. Not all of these schools appear to give (as yet) the same priority to progress.^81^ Yet, where these shifts are taking place (with ‘push’ from government and Ofsted imperatives) along with the embedding of digital technology into the classroom, a proliferation of data has been the result. This has profound implications for geographies of education and cultural geographies, especially those that consider the relationship between culture and education (and cultures of education). Digital forms of mediation and the experiences of data-based living are not simply layers that can be added on existing accounts of cultural life. In the data-based school, the curricula, the modes of assessment and teacher’s and pupil’s roles are being significantly reshaped to enable evidence-based learning and account giving. The teacher becomes less a transmitter of information but a data producer and analyst who enrols the child as the same – as a social scientist of their own learning ability, achievements and life trajectory. In the school as ‘data factory’ or perhaps better ‘data centre’, the ability to create and maintain, through this data, atmospheres of progress has become critical to producing the successfully schooled subject.

Although grades have long been used to classify and sort, to motivate and shame, through discussion of empirical evidence from school in North East England, this article has suggested that the contemporary data-based school enrols young people in projects of education through the creation and maintenance of collective and individualising affective atmospheres of progress. In this way, I have sought to contribute to theorisations of affective atmospheres in geography and how they come to be known (as a question of both experience and method). These atmospheres are not spontaneous ephemera but draw on data’s significant material presences in the school and the lived experiences of the persons who may find themselves contributing to or disrupting such atmospheres. The data are used strategically but are ambivalent and work is done, although not always successfully, to make data work for the motivation of pupils as maintaining the circulation of feelings of progress. These interpersonal sensibilities remain fragile and contested. This should caution claims that data are ‘doing’ any one thing only in schools (such as dehumanising pupils and teachers). As contingent and contested, the life of data in enabling data-based living is polyvalent and ambivalent and therefore requires more careful empirical research and theorisation.

This article has also advanced a novel theorisation of progress ‘after the affective turn’, which is to say that the progress described here is not sufficiently understood as developmental fact, logic, ideology or discourse, but as felt. Pupils experience these atmospheres of progress, and the encounters with data which support them, in varying ways, only some of which have been explored here. Some express confusion, dejection, motivation, surprise, excitement, shame, nervousness and happiness. Many use the language of ‘push’ to express the double move of being pushed and pushing oneself. It is a language not necessarily of violence but certainly of exertion; this is atmospheres of progress as ‘pockets’ of shared senses of effortful movement and improvement that results in individual and collective good feeling. While some like Dave feel that this can result in people being pushed beyond their ability, others like Nicki try to use prior data to challenge what they experience as educational injustice. To experience a lack of attention and challenge can be to feel abandoned by the school to your own efforts in an uneven geography of ‘push’. Exploring the affective dimensions of progress allows for the extension of the understanding and critique of the nature of ‘projects of progress’ more broadly. It also suggests why such developmentalist critiques may gain little traction, even among those who labour fitfully to produce ‘progress’, where a majority are enveloped in the positive feelings that can arise in such atmospheres of progress.

## Notes

1. M.Power, *The Audit Society: Rituals of Verification* (Oxford: Oxford University Press, 2001); M.Bushnell, ‘Teachers in the Schoolhouse Panopticon: Complicity and Resistance’, *Education and Urban Society*, 35(3), 2003, pp. 251–72; C.Hall and A.Noyes, ‘New Regimes of Truth: The Impact of Performative School Self-Evaluation Systems on Teachers’ Professional Identities’, *Teaching and Teacher Education*, 25, 2009, pp. 850–6; C.Hall and E.Millard, ‘The Means of Correct Training? Teachers, Foucault and Disciplining’, *Journal of Education for Teaching*, 20(2), 1994, pp. 153–60; M.Maguire, J.Perryman, S.Ball and A.Braun, ‘The Ordinary School – What Is It?’, *British Journal of Sociology of Education*, 32(1), 2011, pp. 1–16.

2. K.Facer, *Learning Futures: Education, Technology and Social Change* (Abingdon: Routledge, 2011).

3. Labour Government (led by Tony Blair – May 1997 to June 2007; and Gordon Brown – June 2007 to May 2010) and Coalition Government formed of the Conservative Party and Liberal Democrats (led by David Cameron – May 2012 to present).

4. D.Miliband, *The Annual Leadership Lecture 2003* (Nottingham: National College of School Leadership, 2003).

5. D.Miliband, ‘Personalised Learning: Building a New Relationship with Schools’, Paper delivered at the North of England education conference, Belfast, 8 January 2004.

6. J.Perryman, ‘School Leadership and Management after Special Measures: Discipline without the Gaze?’, *School Leadership & Management*, 25(3), 2005, pp. 281–97; Hall and Noyes, ‘New Regimes of Truth’.

7. G.Rose, ‘Interface, Network and Friction: Cultural Geography and Digital Technologies’, *Progress in Human Geography*, in press. See also G.Rose, ‘Cultural Geography and the Digital: What’s Been Written by Cultural Geographers?’, 2014, <http://visualmethodculture.wordpress.com/2014/09/17/cultural-geography-and-the-digital-whats-been-written-by-cultural-geographers/> (20 September 2014).

8. See, for example, ‘School Effectiveness and School Improvement: An International Journal of Research, Policy and Practice’ published since 1990, an indicative example of this work being M.C.M.Ehren and M.S.L.Swanborn, ‘Strategic Data Use of Schools in Accountability Systems’, *School Effectiveness and School Improvement: An International Journal of Research, Policy and Practice*, 23(2), 2012, pp. 257–80. Other work in this area has often reflected on what could be done, some as ‘how to’ manuals with less empirical material on how these changes are playing out in practice. This is changing though; see, for example, the edited text by Schildkamp and others. L.Earl and S.Katz, ‘Leading Schools in a Data Rich World’, <http://www.eed.state.ak.us/nclb/2008wc/Focus_On_Leadership.pdf> (5 March 2012); L.Earl and S.Katz, *Leading Schools in a Data Rich World: Harnessing Data for School Improvement* (London: SAGE, 2006); A.Kelly, C.Downey and W.Rietdijk, *Data Dictatorship and Data Democracy: Understanding Professional Attitudes to the Use of Pupil Performance Data in English Secondary Schools* (Reading: CfBT Education Trust, 2010), <http://www.cfbt.com/en-GB/Research/Research-library/2010/r-data-dictatorship-2010> (23 January 2012); A.Kelly and C.Downey, *Using Effectiveness Data for School Improvement: Developing and Utilising Metrics* (London: Routledge, 2011); K.Schildkamp, M.K.Lai and L.Earl (eds), *Data-Based Decision Making in Education: Challenges and Opportunities* (London: Springer, 2013).

9. Some emerging work from educationalists in a more critical vein can be seen in K.Facer, ‘Personal, Relational and Beautiful: Education, Technologies and John Macmurray’s Philosophy’, *Oxford Review of Education*, 38, 2012, pp. 709–25; S.Ball, M.Maguire, A.Braun, J.Perryman and K.Hoskins, ‘Assessment Technologies in Schools: “Deliverology” and the “Play of Dominations”’, *Research Papers in Education*, 27(5), 2012, pp. 513–33.

10. M.Lawn (ed.), *The Rise of Data in Education Systems: Collection, Visualisation and Uses* (Oxford: Symposium Books, 2013).

11. This is acknowledged by the following pieces, but these tend to focus on high-stakes tests and the places of these as generating ‘labels’ based on absolute achievement rather than data more broadly. J.Booher-Jennings, ‘Learning to Label: Socialisation, Gender, and the Hidden Curriculum of High-Stakes Testing’, *British Journal of Sociology of Education*, 29(2), 2008, pp. 149–60; D.Carless and R.Lam, ‘The Examined Life: Perspectives of Lower Primary School Students in Hong Kong’, *Education 3–13: International Journal of Primary, Elementary and Early Years Education*, 42(3), 2014, pp. 313–29; D.Reay and D.Wiliam, ‘“I’ll be a Nothing”: Structure, Agency and the Construction of Identity through Assessment’, *British Educational Research Journal*, 25(3), 1999, pp. 343–54.

12. Kelly and Downey, *Using Effectiveness Data*, p. 416.

13. While the school has given me permission to identify them, I have chosen not to in this article, and teachers are included in the article left unnamed. Pupil’s names are the pseudonyms they chose.

14. The school serves an area that is in the most deprived wards in County Durham and in the most deprived 5 per cent of Lower layer Super Output Area (LSOA) in England (2010).

15. C.J.Mitchell, ‘Case and Situation Analysis’, *Sociological Review*, 31, 1956, pp. 187–211.

16. B.Flyverg, ‘Five Misunderstandings about Case-Study Research’, *Qualitative Inquiry*, 12(2), 2006, 219–45.

17. See Note 30.

18. See, for example, Jöns and Hoyler, ‘Global Geographies of Higher Education’, pp. 45–59; J.Pykett, ‘Making Citizens in the Classroom: An Urban Geography of Citizenship Education?’, *Urban Studies* 46(4), 2009, pp. 803–23.

19. J.Kenway and D.Youdell, ‘The Emotional Geographies of Education: Beginning a Conversation’, *Emotion, Space and Society*, 4, 2011, pp. 131–6.

20. This position is engaged with by Facer in ‘Personal, Relational and Beautiful’.

21. E.Uprichard, ‘Children as ‘Being and Becomings’: Children, Childhood and Temporality’, *Children & Society*, 22, 2008, 303–13.

22. D.Chakrabarty, *Provincializing Europe: Postcolonial Thought and Historical Difference* (Princeton: Princeton University Press, 2000), p. 224.

23. L.Edelman, *No Future: Queer Theory and the Death Drive* (London: Duke University Press, 2004), p. 2ff.

24. D.Archard, *Children: Rights and Childhood* (London: Routledge, 1993).

25. C.Katz, ‘Accumulation, Excess, Childhood: Toward a Countertopography of Risk and Waste’, *Documents d’Anàlisi Geogràfica*, 57(1), 2011, pp. 47–60.

26. C.H.Thiem, ‘Thinking through Education: The Geographies of Contemporary Educational Restructuring’, *Progress in Human Geography*, 33(2), 2009, pp. 154–73.

27. See Note 1.

28. Kelly et al., *Data Dictatorship and Data Democracy*, p. 5; Kelly and Downey, *Using Effectiveness Data*; Earl and Katz, *Leading Schools in a Data Rich World*.

29. L.Croxford, J.Gray and J.Ozga, ‘Teacher Attitudes to Quality Assurance and Evaluation (QAE) in Scotland and England’, CES Briefing, No. 51 (Edinburgh: Centre for Educational Sociology, The University of Edinburgh, September 2009), pp. 1–4; Kelly et al., *Data Dictatorship and Data Democracy*; Kelly and Downey, *Using Effectiveness Data*.

30. Pupil with pupil (peer marking or name calling), pupil with teacher (survey feedback given about staff), teacher with pupil (marking and grade giving), teacher with teacher (lesson observations), head teacher with teacher (performance management processes), local and national government with schools (local oversight and support; Ofsted, the independent schools inspectorate; league tables) and internationally through comparisons such as PISA (the OECD Programme for International Student Assessment) See Figure 1 in Ball et al., ‘Assessment Technologies in Schools’ and on the wider scales Croxford et al., ‘Teacher Attitudes’; S.Grek and J.Ozga, ‘Governing by Numbers? Shaping Education through Data’, CES Briefing, No. 44 (Edinburgh: Centre for Educational Sociology, The University of Edinburgh, February 2008), pp. 1–4; M.Lawn and J.Ozga, ‘The Sleep of Reason Breeds Monsters: Data and Education Governance in England’, CES Briefing, No. 52, (Centre for Educational Sociology, The University of Edinburgh, Edinburgh, September 2009), pp. 1–4.

31. J.Ozga, P.Dahler-Larsen, C.Segerholm and H.Simola (eds), *Fabricating Quality in Education: Data and Governance in Europe* (London: Routledge, 2011).

32. B.Williamson, ‘Reassembling Children as Data Doppelgangers: How Databases Are Making Education Machine-Readable’, Powerful Knowledge conference, University of Bristol, Bristol, 16 May 2014.

33. N.Selwyn, ‘Data Entry: Towards the Critical Study of Digital Data and Education’, *Learning Media and Technology*, 40, 2015, 64–82.

34. Secondary schooling begins in England typically from the year (starting in September) in which the child turns 12 years old. This is known as Year 7.

35. This vignette is drawn from ethnographic notes, and the young people’s words in this section are not verbatim.

36. B.Anderson, *Encountering Affect: Capacities, Apparatuses, Conditions* (Dorchester: Ashgate, 2014).

37. S.Bragg and H.Manchester, *Creativity, School Ethos and the Creative Partnerships programme* (Newcastle: Creativity, Culture and Education, 2011); H.Manchester and S.Bragg, ‘School Ethos and the Spatial Turn: “Capacious” Approaches to Research and Practice’, *Qualitative Inquiry*, 19(10), 2013, pp. 818–27.

38. B.Anderson, ‘Affective Atmospheres’, *Emotion, Space and Society*, 2, 2009, p. 77.

39. P.Adey, L.Brayer, D.Masson, P.Murphy, P.Simpson and N.Tixier, ‘“Pour votre tranquillité”: Ambiance, Atmospheres, and Surveillance’, *Geoforum*, 49, 2013, pp. 299–309.

40. Anderson, ‘Affective Atmospheres’, p. 78.

41. K.Stewart, ‘Atmospheric Attunements’, *Environment and Planning D: Society and Space*, 2011, 29(3), 445–53.

42. A selection of recent work drawn on for this section more broadly includes Adey et al., ‘“Pour votre tranquillité”, pp. 299–309; Anderson, ‘Affective Atmospheres’; C.Hemmings, ‘In the Mood for Revolution: Emma Goldman’s Passion’, *New Literary History*, 43(3), 2012, pp. 395–417; D.Bissell, ‘Passenger Mobilities: Affective Atmospheres and the Sociality of Public Transport’, *Environment and Planning D: Society and Space*, 28(2), 2010, pp. 270–89; T.Edensor, ‘Illuminated Atmospheres: Anticipating and Reproducing the Flow of Affective Experience in Blackpool’, *Environment and Planning D: Society and Space*, 30, 2012, pp. 1103–22; D.McCormack, ‘Governing Economic Futures through the War on Inflation’, *Environment and Planning A*, 44, 2012, pp. 1536–53; D.McCormack, ‘Geographies for Moving Bodies: Thinking, Dancing, Spaces’, *Geography Compass* 2(6), 2008, pp. 1822–36; D.McCormack, ‘Engineering Affective Atmospheres on the Moving Geographies of the 1897 Andrée Expedition’, *Cultural Geographies*, 15, 2008, pp. 413–30; Stewart, ‘Atmospheric Attunements’.

43. Bissell, ‘Passenger Mobilities’, p. 273.

44. Stewart, ‘Atmospheric Attunements’.

45. Anderson, ‘Affective Atmospheres’, pp. 79–80.

46. Anderson, ‘Affective Atmospheres’, p. 80.

47. Bissell, ‘Passenger Mobilities’, p. 272.

48. Stewart, ‘Atmospheric Attunements’, p. 446.

49. S.Ahmed, *The Promise of Happiness* (Durham: Duke University Press, 2010).

50. Ahmed, *The Promise of Happiness*, pp. 65–6.

51. From year 7 to year 11, that is from 11- and 12-year-olds who in the English system have begun secondary education to 15- and 16-year-olds who are finishing compulsory education. The school does not have provision for pupils to take further qualifications after they are 16 – they go elsewhere.

52. See Note 74.

53. Kelly et al., *Data Dictatorship and Data Democracy*; Kelly and Downey, *Using Effectiveness Data for School Improvement*.

54. ‘Student Voice’ often comprises of the representative function of a student council with elected members but may also involve undertaking projects with research and action components. See, for example, J.Rudduck and M.Fielding, ‘Student Voice and the Perils of Popularity’, *Educational Review*, 2006, 58(2), pp. 219–31.

55. See Note 3.

56. See Note 3.

57. H.Goldstein, J.Rasbash, M.Yang, G.Woodhouse, H.Pan, D.Nuttall and S.Thomas, ‘A Multilevel Analysis of School Examination Results’, *Oxford Review of Education*, 1993, 19(4), pp. 425–33.

58. A.Llewellyn, ‘Progress – Is It Worth It? A Discussion of Productions of Progress in Mathematics Education’, Mathematics Education and Contemporary Theory conference paper, 2013, <http://www.esri.mmu.ac.uk/mect2/papers_13/llewellyn.pdf> (15 August 2013) and Department for Education and Employment (DfEE) *Excellence in Schools* (London: DfEE, 1997).

59. Goldstein et al., ‘A Multilevel Analysis of School Examination Results’; J.Rasbash, G.Leckie and R.Pillinger, ‘Children’s Educational Progress: Partitioning Family, School and Area Effects’, *Journal of the Royal Statistical Society*, 173(3), 2010, pp. 657–82.

60. See Note 3; Department for Education, *Teachers’ Standards*, 2013.

61. Reay and Wiliam, ‘I’ll be a Nothing’; Ball et al., ‘Assessment Technologies in Schools’.

62. R.B.Barr and J.Tagg, ‘From Teaching to Learning: A New Paradigm for Undergraduate Education’, *Change*, 27(6), 1995, pp. 13–25.

63. M.Schweisfurth, *Learner-Centred Education in International Perspective: Whose Pedagogy for Whose Development?* (Abingdon: Routledge, 2013).

64. See also A.Allen, ‘Cultivating the Myopic Learner: The Shared Project of High-Stakes and Low-Stakes Assessment’, *British Journal of Sociology of Education*, 33(5), 2012, p. 653.

65. Or ‘dividualised’ see G.Deleuze, ‘Postscript on the Societies of Control’, *October*, 59, 1992, pp. 3–7.

66. Ofsted, ‘Press Release: Ofsted Announces Changes to Inspections of Schools, Further Education and Skills, and Initial Teacher Education’, 2012, <http://www.ofsted.gov.uk/news/ofsted-announces-changes-inspections-of-schools-further-education-and-skills-and-initial-teacher-edu> (30 May 2012).

67. P.Freire, *Pedagogy of the Oppressed* (London: Penguin, 1996 [1972]).

68. See also M.Watkins, ‘Teachers’ Tears and the Affective Geography of the Classroom’, *Emotion, Space and Society*, 4, 2011, pp. 137–43.

69. M.Finn and C.McEwan, ‘Left in the Waiting Room of History? Provincialising the European Child’, *Interventions: International Journal of Postcolonial Studies*, 17(1), 2015, pp. 113–134.

70. Finn and McEwan, ‘Waiting Room’.

71. Chakrabarty, *Provincializing Europe*.

72. Chakrabarty, *Provincializing Europe*, p. 4.

73. While it goes beyond the scope of this article to explore how the capacities to read and respond to the individual and collective affective life of pupils are learnt and habituated, a body of ‘how to’ and academically informed literature has been developed in education regarding this most commonly under the term ‘behaviour management’. See, for example, the work of Bill Rogers and Sue Cowley.

74. P.Willis, *Learning to Labour: How Working Class Kids Get Working Class Jobs* (Aldershot: Gower, 1981); A.Lareau and E.B.Weininger, ‘Cultural Capital in Educational Research: A Critical Assessment’, *Theory and Society*, 32, 2003, pp. 567–606; A.Lareau, ‘Social Class Differences in Family-School Relationships: The Importance of Cultural Capital’, *Sociology of Education*, 60(2), 1987, pp. 73–85; A.Lareau and E.M.Horvat, ‘Moments of Social Inclusion and Exclusion Race, Class, and Cultural Capital in Family-School Relationships’, *Sociology of Education*, 72(1), 1999, pp. 37–53; P.DiMaggio, ‘Cultural Capital and School Success: The Impact of Status Culture Participation on the Grades of U.S. High School Students’, *American Sociological Review*, 47(2), 1982, pp. 189–201.

75. G.Brown, ‘Emotional Geographies of Young People’s Aspirations for Adult Life’, *Children’s Geographies*, 9(1), 2011, pp. 7–22; S.Holloway and H.Pimlott-Wilson, ‘The Politics of Aspiration: Neo-Liberal Education Policy, ‘Low’ Parental Aspirations, and Primary School Extended Services in Disadvantaged Communities’, *Children’s Geographies*, 9(1), 2011, pp. 79–94.

76. Michael Gove, the UK minister for the Department of Education at the time of research, brought in changes which could be characterised as a return to a focus on ‘traditional’ subjects, an increased focus on facts in relation to skills and a return to terminal rather than modular examinations.

77. See Note 75.

78. A pathway is a timetable based on a set of subjects being studied. As a ‘package’, some pupils find certain elements too easy or too hard based on their particular sense of competence in that subject.

79. Ball et al., ‘Assessment Technologies in Schools’.

80. This is in contrast to the section called ‘“Necessary interventions” (Nicola, Atwood, English) – working on the C/D boundary’ in Ball et al., ‘Assessment Technologies in Schools’.

81. Ball et al., ‘Assessment Technologies in Schools’ suggest a mixed picture of multiple foci.

